# Accuracy of Intra-Oral Radiography and Cone Beam Computed Tomography in the Diagnosis of Buccal Bone Loss

**DOI:** 10.3390/jimaging9080164

**Published:** 2023-08-17

**Authors:** Véronique Christiaens, Ruben Pauwels, Bassant Mowafey, Reinhilde Jacobs

**Affiliations:** 1 Department of Periodontology and Oral Implantology, Faculty of Medicine and Health Sciences, Dental School, Ghent University, C. Heymanslaan 10, 9000 Ghent, Belgium; 2 Department of Dentistry and Oral Health, Aarhus University, 8000 Aarhus, Denmark; ruben.pauwels@dent.au.dk; 3 OMFS IMPATH, Department of Imaging & Pathology, Faculty of Medicine, KU Leuven and Maxillofacial Surgery, University Hospitals Leuven, 3000 Leuven, Belgium; bassantmowafey@hotmail.com; 4 Department of Radiology, Chulalongkorn University, 10330 Bangkok, Thailand; 5 Department of Oral medicine, Periodontology, Diagnosis and Oral radiology, Faculty of Dentistry, Mansoura University, 35516 Mansoura, Egypt; 6 Department of Dental Medicine, Karolinska Institutet, 14152 Huddinge, Sweden

**Keywords:** intra-oral radiography, cone beam CT, diagnosis, buccal bone level

## Abstract

Background: The use of cone beam computed tomography (CBCT) in dentistry started in the maxillofacial field, where it was used for complex and comprehensive treatment planning. Due to the use of reduced radiation dose compared to a computed tomography (CT) scan, CBCT has become a frequently used diagnostic tool in dental practice. However, published data on the accuracy of CBCT in the diagnosis of buccal bone level is lacking. The aim of this study was to compare the accuracy of intra-oral radiography (IOR) and CBCT in the diagnosis of the extent of buccal bone loss. Methods: A dry skull was used to create a buccal bone defect at the most coronal level of a first premolar; the defect was enlarged apically in steps of 1 mm. After each step, IOR and CBCT were taken. Based on the CBCT data, two observers jointly selected three axial slices at different levels of the buccal bone, as well as one transverse slice. Six dentists participated in the radiographic observations. First, all observers received the 10 intra-oral radiographs, and each observer was asked to rank the intra-oral radiographs on the extent of the buccal bone defect. Afterwards, the procedure was repeated with the CBCT scans based on a combination of axial and transverse information. For the second part of the study, each observer was asked to evaluate the axial and transverse CBCT slices on the presence or absence of a buccal bone defect. Results: The percentage of buccal bone defect progression rankings that were within 1 of the true rank was 32% for IOR and 42% for CBCT. On average, kappa values increased by 0.384 for CBCT compared to intra-oral radiography. The overall sensitivity and specificity of CBCT in the diagnosis of the presence or absence of a buccal bone defect was 0.89 and 0.85, respectively. The average area under the curve (AUC) of the receiver operating curve (ROC) was 0.892 for all observers. Conclusion: When CBCT images are available for justified indications, other than bone level assessment, such 3D images are more accurate and thus preferred to 2D images to assess periodontal buccal bone. For other clinical applications, intra-oral radiography remains the standard method for radiographic evaluation.

## 1. Introduction

Almost every diagnosis in dentistry is based on a combination of a clinical as well as a radiographic examination. Both components are important and often essential for an accurate diagnosis and proper treatment planning. For example, the diagnosis in cases of periodontal disease is based on a careful clinical examination (including pocket depth, bleeding on probing, and clinical attachment level) to identify active periodontal disease, while radiographic examination is essential to demonstrate past disease activity in terms of bone loss [1]. Clinical parameters have a high diagnostic sensitivity, while radiographs show a high diagnostic specificity [2,3]. Diagnostic accuracy is obviously increased when clinical information and radiographic information are pooled [4].

Panoramic radiography is most often used to provide an overview of the overall bone loss and evaluate anatomical structures. However, the diagnostic quality of a panoramic radiograph is heavily dependent on careful attention to technique and processing. Furthermore, the lack of image sharpness and the superimposition of multiple structures can be considered as limitations of panoramic radiography as they may result in an underestimation of bone loss in the early stages of the disease [1,5]. In a controlled study by Åkesson et al. [5], panoramic radiographs were compared to bitewings and intra-oral radiographs in terms of accurately capturing bone levels around natural teeth. All types of radiographs underestimated the true marginal bone level as assessed intra-surgically, especially panoramic radiographs [5,6]. Due to the limitations of panoramic radiography, the use of bitewing radiography or intra-oral radiography can be considered during periodontal examination. Both two-dimensional (2D) radiographic techniques are more suitable for the detection of interdental bone loss as they provide high-resolution images with lower radiation compared to panoramic radiography. Multiple studies [5,6,7] have demonstrated that intra-oral radiographs show the least deviation when evaluating interdental bone levels; however, data about buccal bone loss are not included in these studies. Although intra-oral radiographs are more detailed in the evaluation of bone loss levels, the use of the parallel technique is of major importance. The bisecting-angle technique results in a different angle of the X-ray beam and may create a variable amount of bone overlap, although this technique can result in an underestimation of the disease [8]. In a pragmatic approach, multiple authors [9,10,11] recommended taking panoramic radiography as the first step of a periodontal assessment and the use of supplementary intra-oral radiographs to clarify dubious loci. However, clinical experience has shown that a definitive diagnosis is not always evident on a panoramic radiograph and that intra-oral radiography is very often superior. Based on this experience, it is clear that an intra-oral X-ray examination is preferable to panoramic radiography for diagnosing cases of bone loss on the facial aspect of the tooth, more commonly known as buccal bone loss. However, due to the 2D nature of this imaging technique, there is an unavoidable overlap of anatomical structures and a lack of 3D information [1]. Overlap and projection errors often lead to an over- or underestimation of the clinical situation. This is well described for interdental bone levels around teeth as well as around implants [4,12]. Multiple studies have evaluated the diagnosis of interdental bone level (=bone loss in between teeth) using a 2D imaging technique, although there are very few studies focusing on the facial/buccal or lingual aspect [13,14]. Based on the current guidelines of the European Commission [15] for radiographs in periodontal assessment using panoramic radiographs, vertical bitewing and periapical radiographs all have their uses, either alone or in combination. The paralleling technique is always indicated when periapical radiographs are used, as it gives a better geometrical perspective on the periodontal bone compared to the bisecting-angle technique. Advantages of the paralleling technique are minimum elongation/distortion and reduction in distortional effects due to bending of the image receptor used [15]. In cases where conventional 2D radiography fails, cone beam computed tomography (CBCT) may overcome these difficulties due to the elimination of the superimposing factor when using this technique. The use of CBCT in dentistry started in the maxillofacial field, where it was used for complex and comprehensive treatment planning. Due to the use of reduced radiation dose compared to a CT scan, CBCT has become a frequently used diagnostic tool in dental practice. The accuracy of CBCT when compared with intra-oral radiography has justified its use for specific cases that require 3D imaging [16,17]. In the literature, the added value of CBCT during orthodontic treatment has been well described [16,18] for the evaluation of detrimental effects on the supporting alveolar bone after orthodontic treatment and rapid maxillary expansion. In periodontics, CBCT images are important to evaluate buccal bone thickness before and after reconstruction of the buccal bone wall [16,19]. Moreover, the buccal cortical bone regions are often evaluated to avoid the risk of resorption of cortical bone margin in case of immediate implant placement [16]. However, published data on the accuracy of CBCT in the diagnosis of buccal bone levels are limited [7,13,14].

This study aims to compare the accuracy of IOR and CBCT in the diagnosis of the extent of buccal bone loss.

## 2. Materials and Methods

### 2.1. Radiographs

**Figure 1:**
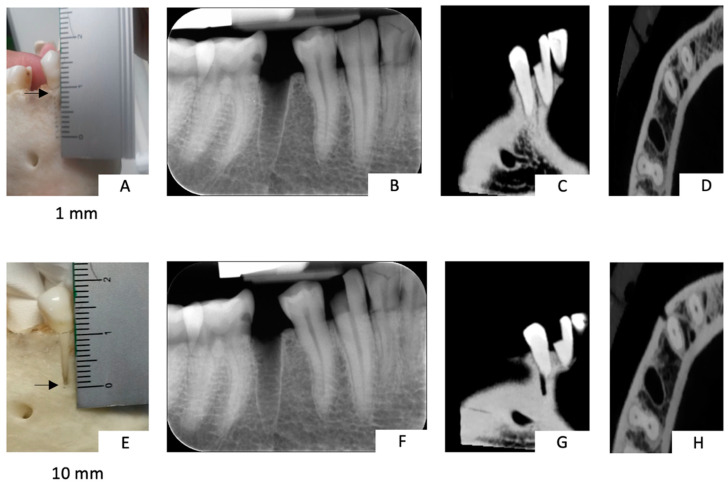
The buccal bone defect was created at the most coronal level of a first premolar in the mandibula and was enlarged apically in steps of 1 mm: (**A**) buccal defect of 1 mm was created; (**B**) periapical radiography and (**C**,**D**) CBCT of the 1 mm defect; (**E**) buccal defect of 10 mm created; and (**F**) periapical radiography and (**G**,**H**) CBCT of the 10 mm defect.

A dry skull was obtained with ethical approval and used to evaluate the accuracy of intra-oral radiography and CBCT in the diagnosis of buccal bone loss around natural teeth. The buccal bone defect was created at the most coronal level of a first premolar in the mandibula and was enlarged apically in steps of 1 mm, starting from a defect of 1 mm and increasing in size up to a defect of 10 mm (Figure 1). After each enlargement, an intra-oral radiograph (Computed Radiography: Digora Optimé, Soredex, Tuusula, Finland) was acquired at 70 kV, 7 mA, and 0.10 s using a Heliodent intra-oral X-ray tube (Sirona, Bensheim, Germany), following a strict paralleling technique [15]. For the radiographic technique used, a parallel X-ray beam was directed perpendicular to the tooth being examined and to the image receptor to provide the best imaging geometry. In addition to this, a bit block was used to help maintain the correct image receptor position relative to the tooth; this was to overcome the risk of the image receptor being bent [15].

At the same time, CBCT (NewTom VGi, Cefla, Verona, Italy) scans were acquired using a field of view of 8 × 5 cm at 110 kV and 15 mAs. The phantom composed of a dry human skull and mandible with natural teeth was positioned in a standard position on a dedicated wooden platform attached to the CBCT positioning device. This set-up allowed free motion and repeated scanning of the phantom with a standard projection geometry. Soft tissue attenuation of the tongue and gingiva was compensated for by adding a removable mix D model, which was composed of a mixture of paraffin wax and other chemicals prepared according to Oenning et al. [20]. All CBCT scans were registered with the elastix software (University Medical Center Utrecht and contributors), using the mutual information metric and a rigid transformation.

Next, two observers (RP and VC) jointly selected three axial slices at different levels of the buccal bone, as well as one transverse slice. Using a macro written in ImageJ, identical slices were extracted from all other CBCT scans and saved as TIFF files.

The study was conducted in accordance with the Declaration of Helsinki of 1975 as revised in 2000. The protocol was approved by the ethical committee of the KU Leuven Research Ethics (NH019-2018-03-02).

### 2.2. Observers

Six dentists with varying specialties participated in the radiographic observations under standard viewing conditions (dimmed room, 80 cm distance from a 48-inch Samsung HR display, Seoul, Republic of Korea). As it was our intention to assess inter-observer variability in the daily clinical practice, the clinicians were deliberately not trained and calibrated beforehand. All measurements were performed independently by each of the observers.

### 2.3. Assessment of Bone Level on the Basis of Radiographs

#### 2.3.1. Buccal Defect Progression

In the first part of the study, the observers received 10 intra-oral radiographs, which registered the bone defect starting from 1 mm up to 10 mm. To overcome errors, the crestal bone margin was marked as a baseline, with a progressive increase in the buccal bone defect with 1 mm steps starting from the baseline. All radiographs were randomly sequenced, and each observer was asked to rank the intra-oral radiographs on the extent of the buccal bone defect, starting with the smallest defect. Afterwards, they repeated the procedure with the CBCT scans based on a combination of axial and transverse information.

#### 2.3.2. Presence or Absence of a Buccal Defect Based on CBCT

For the second part of the study, each observer was asked to evaluate the axial and transverse CBCT slices on the presence or absence of a buccal bone defect. To blind the observers from the nature of the investigation, the aforementioned CBCT slices were randomly pooled with a selection of axial and transverse slices that were different from the slices of the first premolar on which the defect was created.

A 9-point visual analog scale (VAS) was used, in which the endpoints corresponded to absolute certainty regarding the absence or presence of a defect, respectively, and the central point corresponded to complete uncertainty regarding the presence of a defect.

### 2.4. Statistical Analysis

For the first part of the study, a weighted kappa was used to compare the true answers with those given by each observer, using linear weights. Furthermore, the absolute error in ranking the defects from 1 to 10 was calculated and compared with a hypothetical 0 value using the Wilcoxon signed-rank tests. The significance level underwent a Bonferroni correction based on the number of observers (n = 6), yielding a value α = 0.05/6 ≈ 0.0833. The second part of the study, containing only the CBCT images, was evaluated using a receiver operating curve (ROC).

Sensitivity and specificity were calculated based on binarized (lesion/no lesion) scores derived from the VAS results; it should be noted that the exact middle point of the VAS scale was classified as ‘no lesion’.

### 2.5. Case Report: Clinical Analysis

**Figure 2:**
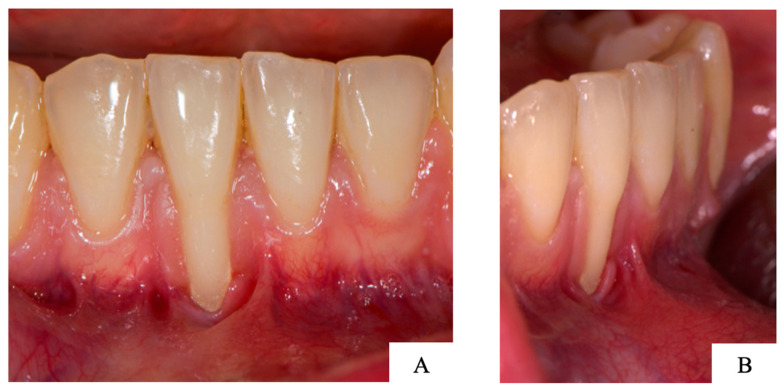
(**A**) Frontal and (**B**) lateral view of the lower incisors with a recession on the right central incisor.

The clinical relevance of this paper can be discussed based on a clinical case of a patient who presented a recession on the right lower central incisor (Figure 2: frontal (A) and lateral (B) view of the clinical presentation). The patient was a non-smoker in a good general health (American Society of Anesthesiologists (ASA) status 1). The patient finished orthodontic treatment a few months earlier and was now referred for recession coverage of the lower incisor.

## 3. Results

### 3.1. Buccal Defect Progression

The kappa values for intra-oral radiography and CBCT are shown in Table 1. On average, the kappa values increased by 0.384 for CBCT compared to intra-oral radiography. Interestingly, three observers showed a negative kappa value for IOR, implying that their ranking of the defects was worse than random scoring (κ = 0). Apart from two observers, who showed equal kappa values for both modalities, CBCT showed superior defect ranking. The average error in ranking as well as the results of the Wilcoxon signed-rank tests for each observer are shown in Table 2. For IOR, four out of six observers showed a significant ranking error, whereas none of the observers showed a significant error for CBCT. The percentage of rankings that were within 1 of the true rank was 32% for IOR and 42% for CBCT.

### 3.2. Presence or Absence of a Buccal Bone Defect Based on CBCT

**Figure 3:**
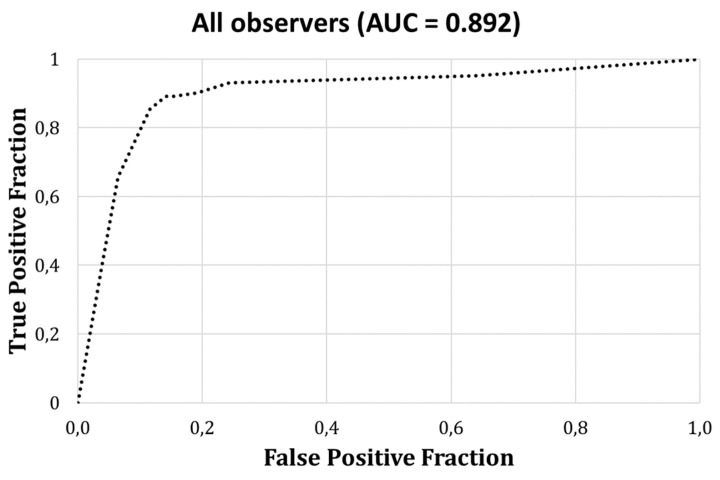
Receiver operating characteristic curve for cone beam computed tomography analysis of buccal bone defects, averaged over all observers. AUC = area under the curve.

**Figure 4:**
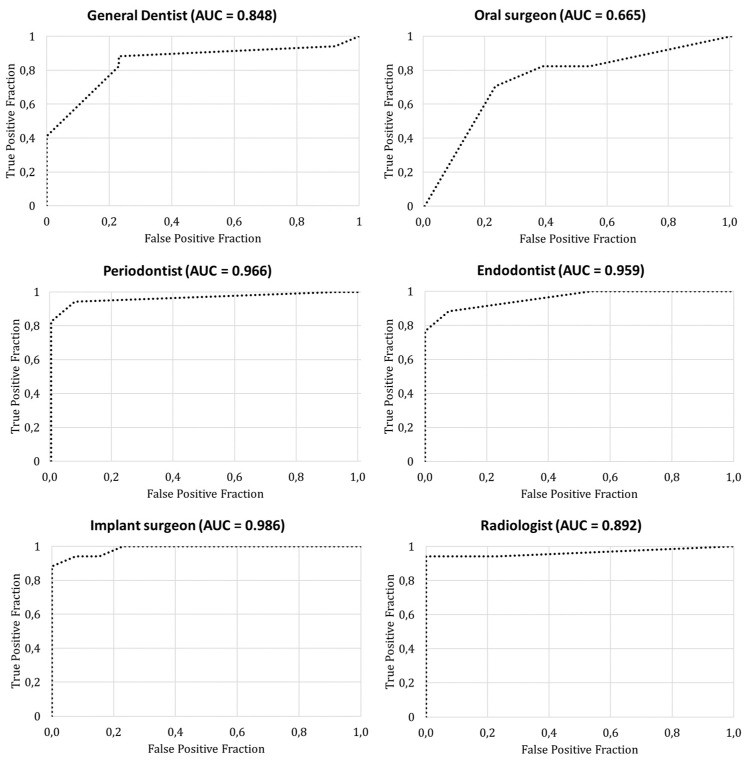
Receiver operating characteristic curve for cone beam computed tomography analysis of buccal bone defects for individual observers. AUC = area under the curve.

The average area under the curve (AUC) of the ROC was 0.892 for all observers (Figure 3). Detailed information of the area under the curve for each observer is presented in Table 3. Three observers showed an AUC above 0.95, whereas the other observers’ AUC ranged between 0.67 and 0.89 (Figure 4).

The overall sensitivity and specificity of CBCT in the diagnosis of the presence or absence of a buccal bone defect was 0.89 and 0.85, respectively. On average, the observers had 78% confidence in their diagnosis; an increased confidence was not related to an increased diagnostic performance.

### 3.3. Case Report: Clinical Analysis

**Figure 5:**
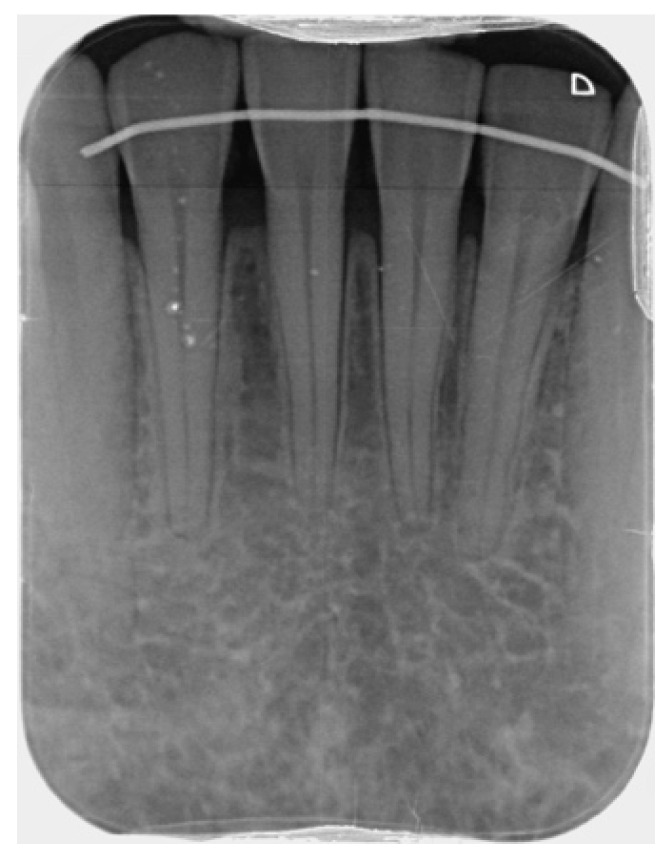
Intra-oral radiograph of the lower incisors.

**Figure 6:**
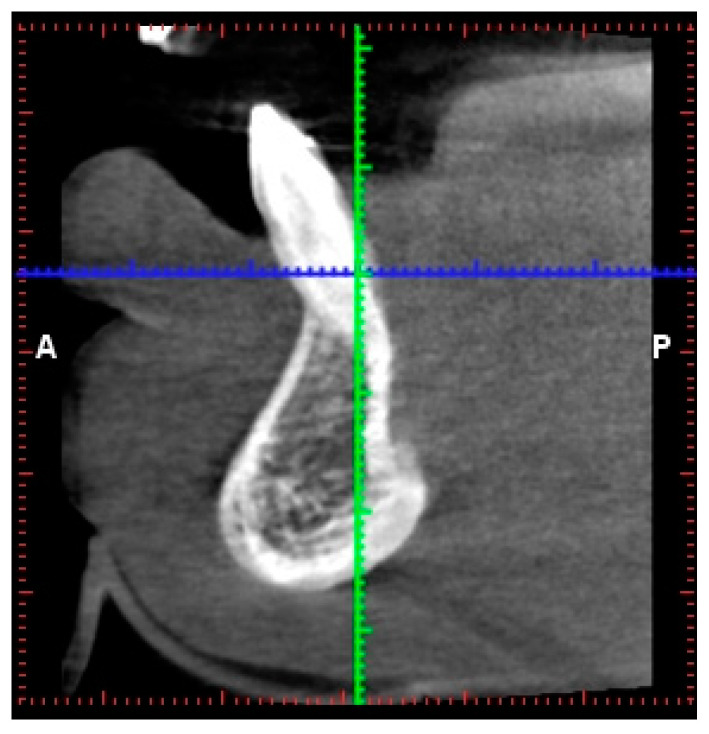
CBCT cross-sectional image of the right lower incisor.

Based on the intra-oral radiograph (Figure 5) of the lower incisors of the patient, no major problem was expected. The interdental bone peaks were within the natural range, and apart from a slightly wider periodontal ligament, this radiograph did not seem to show a disturbing situation. Signs to suggest that the buccal bone around the right lower incisor was absent for more than two-thirds of the root length were absent. The CBCT images (Planmeca, Helsinki, Finland) of the same area reveal unexpected problems with buccal bone loss, which was unable to be diagnosed using the 2D radiograph (Figure 6). Indeed, while no major problem in terms of an absence of buccal bone was expected on the intra-oral radiograph, CBCT clearly illustrated a doubtful prognosis of the right central incisor, with this tooth being anchored for less than 1/3 in the bone.

## 4. Discussion

The current study, along with the case presentation, clearly illustrates the impact of overlapping anatomical structures on an intra-oral radiograph that may lead to a significant radiographic overestimation of the buccal bone level. As intra-oral radiography is a projection in which all absorption occurring along the X-ray path from the source to the detector is mapped as a sum, this can be seen as an inherent limitation of this diagnostic tool. In the literature, overlap of anatomical structures and image distortion are well-described shortcomings of intra-oral radiographs, such as periapical radiographs [21]. Especially for diagnosis of a buccal bone defect, volumetric images with a high dimensional accuracy and no overlap of buccal and lingual/palatal bone are required. CBCT provides 3D information at a high resolution of teeth, surrounding bone, and other anatomical structures [22,23]. In implant dentistry, CBCT seems to be the preferred pre-operative radiographic method due to its high dimensional accuracy [24,25,26]. Keeping these advantages in mind, CBCT provides opportunities for more accurate diagnosis in the dental field.

The usefulness of CBCT for several diagnostic purposes in dental implantology and orthodontics has been well described [27,28]. However, limited studies have been reporting on the advantages of CBCT for periodontal diagnosis, including buccal bone levels [13,17]. Due to overlap of anatomical structures and the absence of 3D information, intra-oral radiographs are unable to diagnose the true distinction between the buccal and lingual cortical plate and complicate the evaluation of periodontal bone defects around teeth and implants [29]. In order to obtain a 3D assessment of bone defects, the current diagnostic approach needs further improvement for early diagnosis of periodontal disease [13,30,31]. Conventional CT could solve this problem by providing axial slices of the object of interest, but it also has some drawbacks, including high radiation dose and higher cost [13,32].

To the best of our knowledge, there is no literature available that discusses the accuracy of IOR and CBCT for the diagnosis of buccal bone defects. The results of the present study show that the interpretability of buccal bone defects differs between IOR and CBCT. The overall reliability for diagnosis of a buccal bone defect is low for IOR and high for CBCT. In other words, it is far more likely to underestimate a buccal bone defect using IOR compared to CBCT. One of the only articles that is similar to our research is the paper by Ruetters et al. [33]. In this pilot study, a low-dose CBCT was used to evaluate the buccal bone adjacent to the mandibular anterior teeth. Both projects demonstrate a superior accuracy of periodontal buccal bone level diagnosis using CBCT (3D technique) compared to intra-oral radiography (2D technique). CBCT provides users with an overall view and allows a quick and detailed evaluation of the buccal bone level, which is impossible using 2D radiography.

Among the observers, the highest average confidence was found for the oral surgeon (95%) and the radiologist (94%), although they showed the worst and third worst diagnostic performance, respectively, based on the AUC. This indicates that diagnostic confidence does not translate to diagnostic efficacy as it is based more on a personal trait that cannot be compared directly between observers. While sensitivity, specificity, and other diagnostic metrics can be objectively defined and used to evaluate and optimize diagnostic processes, confidence-related metrics should be used with care. Ideally, confidence should only be considered in diagnostic research as a comparative metric for the same person or a group of people (e.g., an observer’s or a group of observers’ confidence before and after receiving additional information), rather than a way to intercompare observers.

While the diagnostic benefit of CBCT over 2D radiography is demonstrable, one should also consider radiation risk [34]. An additional consideration is the wide range in image quality and radiation dose between CBCT machines, or between different exposure protocols of a CBCT unit. The imaging performance of CBCT is dictated by several factors, including, but not limited to, kV, mAs, and voxel size, which are not standardized between manufacturers or clinics [34,35,36].

The use of a cadaver can be seen as a limitation of this study as scatter radiation produced by the soft tissues of a patient might not be exactly the same as the experimental set-up. On the other hand, the ex vivo study set-up allowed for a ROC analysis due to the availability of the ground truth. Furthermore, creating extensive bone defects and taking multiple intra-oral radiographs and CBCT scans in a patient would be ethically unacceptable. For this reason, the methodology used is justified and in line with other studies [13,14,33].

Overall, one should always keep in mind the principles of justification and optimization, with the need to go for indication-oriented and patient-specific imaging always be kept in mind (ALARA/ALADAIP principle) [37,38]. Considering the ongoing evolution in CBCT imaging (lower radiation dose, shorter acquisition time, etc.), further studies focusing on CBCT for radiographic bone level assessment should be conducted.

## 5. Conclusions

When CBCT images are present for justified indications other than bone level assessment, the available CBCT images are more accurate than 2D images in assessing buccal periodontal bone and are thus preferred. CBCT should be used as a secondary radiographic tool; for example, in cases with clinical doubts or lacking information of the buccal bone level, an indication-specific CBCT can also be justified to avoid improper treatment planning and complications at outcome. For other clinical applications, intra-oral radiography remains the standard method for radiographic evaluation.

## Figures and Tables

**Table 1:** Kappa values for each observer for intra-oral radiography (IOR) and cone beam computed tomography (CBCT). Average values among observers are shown in bold.

Observer	IOR vs. Ground Truth	CBCT vs. Ground Truth
General dentist	−0.273	0.212
Oral surgeon	−0.152	0.818
Periodontist	0.394	0.394
Endodontist	0.515	0.515
Implant surgeon	−0.333	0.333
Radiologist	0.212	0.394
**Average**	**0.061**	**0.444**

**Table 2:** Average error from the true rank of defect severity for intra-oral radiography (IOR) and cone beam computed tomography (CBCT), and percentage of rankings within 1 of the true rank. Asterisks (*) denote a significant average error. Average values among observers are shown in bold.

Observer	IOR		CBCT	
	Avg. Error	% with Error ≤ 1	Avg. Error	% with Error ≤ 1
General dentist	4.2	10%	2.6	30%
Oral surgeon	3.8 *	30%	0.6	80%
Periodontist	2.0 *	60%	2.0	30%
Endodontist	1.6	60%	1.6	40%
Implant surgeon	4.4 *	0%	2.2	30%
Radiologist	2.6 *	30%	2.0	40%
**Average**	**3.1**	**32%**	**1.8**	**42%**

**Table 3:** Receiver operating characteristic curve’s area under the curve (AUC), sensitivity, and specificity for each observer, along with average confidence according to the VAS results.

Observer	AUC	Sensitivity	Specificity	Average Confidence (%)
General dentist	0.848	0.82	0.77	68
Oral surgeon	0.665	0.82	0.62	95
Periodontist	0.966	0.94	0.92	73
Endodontist	0.959	0.88	0.92	53
Implant surgeon	0.986	0.94	0.85	85
Radiologist	0.892	0.94	1.00	94

## Data Availability

The data presented in this study are available on request from the corresponding author. The data are not publicly available due to ethical restrictions.
